# Is otologic surgery contributing to the opioid epidemic?

**DOI:** 10.1186/s40463-021-00521-1

**Published:** 2021-06-22

**Authors:** Valerie Dahm, Justin T. Lui, Rudolfs Liepins, Joseph M. Chen, Trung N. Le, Christoph Arnoldner, Vincent Y. W. Lin

**Affiliations:** 1 Department of Otolaryngology–Head & Neck Surgery, Sunnybrook Health Sciences Centre, University of Toronto, 2075 Bayview Ave, Toronto, ON M4N 3M5 Canada; 2 Department of Otorhinolaryngology, Head & Neck Surgery, Medical University of Vienna, Waehringer Guertel 18-20, 1090 Vienna, Austria

**Keywords:** Otologic surgery, Pain, Opioids, Prescription, Medication

## Abstract

**Background:**

The opioid epidemic is a significant public health crisis challenging the lives of North Americans. Interestingly, this problem does not exist to the same extent in Europe. Surgeons play a significant role in prescribing opioids, especially in the context of post-operative pain management. The aim of this study was to compare the post-surgical prescribing patterns of otologists comparing Canada and Austria.

**Methods:**

An online questionnaire was sent to 33 Canadian and 32 Austrian surgeons, who perform otologic surgery on a regular basis. Surgeons were asked to answer some questions about their background as well as typical prescribing patterns for postoperative pain medication for different ear surgeries (cochlear implant, stapedotomy, tympanoplasty). In addition, surgeons were asked about the typical use of local anesthetics for pain control at the end of a procedure. Otologists gave an estimate how confident they were that their therapy and prescriptions provide sufficient pain control to their patients.

**Results:**

Analysis of the returned questionnaires showed that opioids are more commonly prescribed in Canada than in Austria. Nonsteroidal anti-inflammatory drugs are used for postoperative pain more regularly after ear surgery in Austria, as opposed to Canada. Some of the prescribed drugs by European otologists are not available in North America. The use of local anesthetics at the end of surgery is not common in Austria. Surgeons´ confidence that the prescribed pain medication was sufficient to control postoperative symptoms was higher in the group not prescribing opioids than in the group that did routinely prescribe opioids.

**Conclusion:**

Prescribing patterns differ substantially between the two evaluated countries. This data suggests an opportunity to reduce opioid prescriptions after otologic surgeries. Studies to evaluate pain after these operations as well as efficacy of analgesics following ear surgery are an important next step.

**Graphical abstract:**

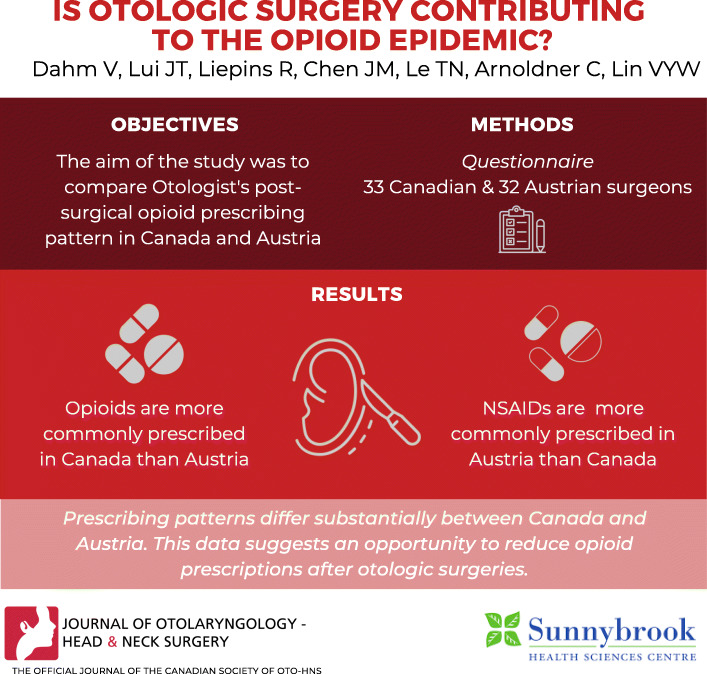

## Background

The opioid epidemic is an evolving public health crisis with overdose-related deaths rising [1]. Surgeons have been linked to this crisis as many opioid prescriptions are given for acute postoperative pain [2] and are often prescribed in excess [3]. Patients do not dispose of unused drugs safely [4] which is an important diversion point, as these medications are also obtained from friends and family [5]. Since there are no guidelines for postoperative pain management after otolaryngologic procedures, pain medication prescribing patterns differ substantially [6]. Interestingly, the problem with overprescribing opioids does not exist to the same extent in Europe [7]. A study comparing prescribing patterns between the U.S., Canada and Sweden concluded that there is a 7-fold higher rate of opioid prescription filled in the immediate postoperative period in North America in comparison to Sweden [8]. A further study on orthopedic trauma showed that there is a greater number of opioids prescribed at discharge in the U.S. compared to Haiti and the Netherlands [9]. There have been studies assessing pain medication following otolaryngologic surgery focusing on opioid prescription patterns and consumption alone [2, 10]. Ngombu et al. assessed opioid tablet use via a postoperative telephone questionnaire and found that while a mean of 17.8 pills were prescribed, only a mean of 7.9 were taken [2]. Mahairas et al. analyzed opioid prescription fulfillment through the national pharmaceutical database [10]. They divided patients into two groups: group 1 filled only one prescription and group 2 filled more than one prescription in the 12 months following surgery. Recurrent opioid use was associated with ‘total morphine milligram equivalents prescribed per day, post-operative chronic pain disorder, post-operative substance abuse and post-operative anxiety. A common conclusion of the above mentioned two studies was that opioid prescription should be limited after otolaryngologic surgery. Nguyen et al. performed a randomized single-blinded trial comparing ibuprofen and opioid based primary analgesic therapy in 108 patients undergoing various otolaryngologic surgeries (30% thyroidectomy, 19% parathyroidectomy, 23% FESS, 9% endolaryngeal procedures, 11% septoplasty or septorhinoplasty and 8% otologic surgery) [11]. Results showed that ibuprofen used as primary therapy can significantly reduce opioid consumption (2.04 tablets/pills versus 4.86). Since pain scores did not differ between groups, authors also concluded that Ibuprofen provides equally effective pain control as hydrocodone/acetaminophen, which was the opioid combination used in this study [11]. The increasing national dialogue and the supposed discrepancy to European countries as well as the scarce data on opioid prescriptions and use of non-steroidal anti-inflammatory drugs (NSAID) after otologic surgery was the impetus behind the creation of this study. We assessed prescription patterns in Canada and compared them to those in Austria following cochlear implantation, stapedotomy, tympanoplasty and tympanomastoidectomy.

## Methods

An online survey via SurveyMonkey Inc. (San Mateo, USA) was created and assessed by several Otolaryngologists for clarity in English and German. The questionnaire consisted of four subsections targeting surgical practice of otololaryngologists. The first section included three basic questions about the background of the answering surgeon (see Table 1). There were three further subsections of different types of otologic surgery: cochlear implant surgery, stapedotomy and tympanoplasty (+/−mastoidectomy). If surgeries using different possible approaches (transcanal versus endaural versus postauricular) were addressed, surgeons were then asked which of their preferred approaches would determine the effect on postoperative pain control. Surgeons were asked to select all medications regularly prescribed after each surgery. Additionally, surgeons were queried about the use of local anesthetic at the end of surgery. At the end of each subsection, surgeons were asked to rate their confidence in their prescribed analgesia protocol from 0 to 10 (scale in steps of whole numbers). In total, the questionnaire was sent to 33 Canadian and 32 Austrian otolaryngologists, with a response rate of 55% (*n* = 18) and 59% (*n* = 19), respectively. Four answer sets were excluded due to missing answers. Consequently, 33 answer sets were used for analysis. Details of the respondents are given in Table 1.

**Table 1.** Demographic representation of surveyed otolaryngologists in Canada and Austria

	Canada	Austria	Total
Questionnaires - sent	33	32	65
Questionnaires - response	18	19	37
Questionnaires - analyzed	16	17	33
Sex			
Male	13	15	33
Female	3	2	
Academic/public hospital	15	17	32
Years of independent practice			
≤ 5 years	3	0	3
6–10 years	5	3	8
11–15 years	3	4	7
16–20 years	1	2	3
> 20 years	4	8	12

Table 1 Column one shows details of the questionnaires sent to Canadian otologist, Column two the details of the questionnaires sent to Austrian otologist, Column 3 (Total) shows a summary of all results. Details show the sex of the otologists, as well as the area in which they work and the years of independent practice

In general, most otologic surgery is performed as day surgery in Canada. In Austria most surgeries are associated with at least one or even two overnight stays at the hospital after surgery. We asked surgeons to provide pain medication given to patients as inpatient and outpatients. We did not assess if the pain medication was given orally or intravenously during hospital stay.

### Statistical analysis

Percentages of surgeons prescribing opioids or not-prescribing opioids were calculated by adding all pain medications given per surgeon (Fig. 2). To calculate variation of pain medications, only surgeons who perform at least two of the surgeries and therefore gave at least two different answers were included. Surgeons were grouped to the variation group (Fig. 3), if they gave different medications for at least one surgery compared to one other.

For the dot diagram (Fig. 4), means of all confidences given by each surgeon were used, which were from two or three ratings. If surgeons only perform one of the surgeries, then only one rating was used. Again, surgeons were grouped in the opioid group, if they gave opioids for any surgery and in the non-opioid group, if they never gave opioids. Significant difference of means was calculated using the Mann-Whitney U test.

## Results

Thirty-four questionnaires were included for statistical analysis. Eight Canadian and 12 Austrian surgeons provided answers about cochlear implant surgery. Thirteen Canadian and 16 Austrians answered questions about stapedotomy. 48% of all surgeons preferred to perform this surgery endaurally (*n* = 14), 41% transcanal (*n* = 12) and 7% (*n* = 2) seemed to perform both approaches to the same extent. Thirty-one surgeons provided answers about tympanoplasty and cholesteatoma surgery. Tympanoplasty for tympanic membrane perforation and ossiculoplasty are mainly performed endaurally in this surgeon group (*n* = 14, 45%). Five surgeons perform these operations via a transcanal approach, with four of them practicing in Canada. Nine otologists performed tympanoplasty for ear drum perforation or ossicular chain reconstruction via a postauricular approach of which six practice in Austria. One Canadian surgeon preferred endoscopic surgery. Small cholesteatomas (e.g. attic cholesteatoma) are mainly done via an endaural approach (*n* = 16, 52%). Large Cholesteatomas are performed by all but one surgeon via a postauricular approach. One otologist mainly completes endoscopic surgeries in patients with this disease. Details are listed in Table 2.

**Table 2.** Surgical approaches

Surgery	Number/Approach	Canada	Austria	Total
Cochlear implantation	N =	8	12	20
Stapedotomy	N =	13	16	29
	Transcanal	62%	31%	45%
	Endaural	31%	63%	48%
	Endaural and transcanal	0%	6%	3%
	Unknown	8%	0%	3%
Tympanoplasty	N =	13	17	31
	Transcanal	31%	6%	16%
	Endaural	38%	53%	45%
	Endoscopic	8%	0%	3%
	Postauricular	23%	35%	29%
	Unknown	0%	6%	3%
Small Cholesteatoma (e.g. attik)	N =	14	17	31
	Transcanal	0%	17%	10%
	Endaural	36%	65%	52%
	Endoscopic	29%	0%	13%
	Postauricular	22%	18%	19%
	Postauricular and endaural	14%	0%	6%
Extensive Cholesteatoma	N =	14	17	31
	Endoscopic	7%	0%	3%
	Postauricular	93%	100%	97%

Table 2: Number of answer sets given per surgery as well as preferred approaches for stapedotomy and for different tympano/mastoidectomies. Tympanoplasty includes tympanic membrane perforation and ossiculoplasty. Some surgeries gave two preferred approaches (e.g. endaural and transcanal)

### Postoperative pain management

**Fig. 1:**
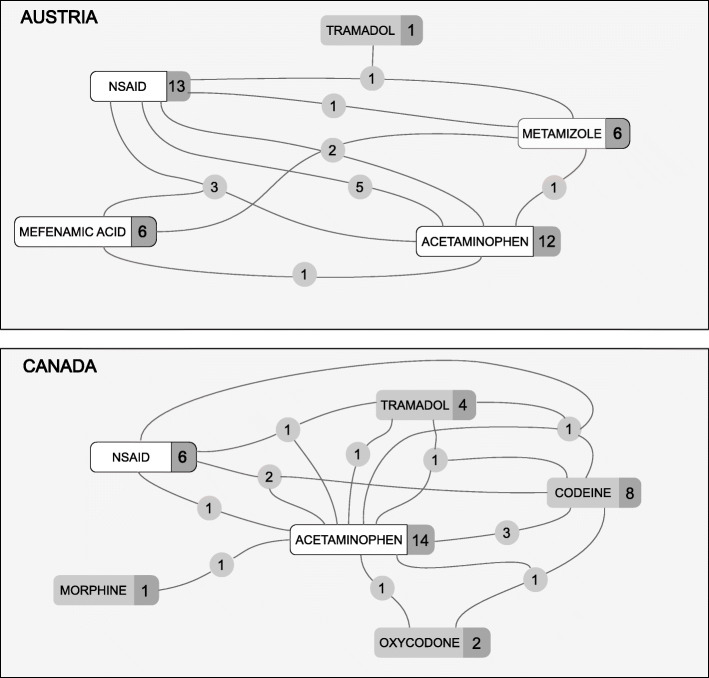
Pain medications given by surgeons. The numbers on the connecting lines indicate how many surgeons choose both these drugs, which might correspond to combinations given. Numbers to the right of the substances indicate how often this medication was picked independent of combinations. Medication with a white background are non-opioids, grey background are opioids. Ibuprofen, diclofenac, naproxen and toradol are combined to NSAID. Mefenamic acid (also an NSAID) was reported separately as it was only given in Austria

Answers on analgesic therapy were given by 15 Canadian and 17 Austrian otologists. All Canadian otologists but one (93%) gave acetaminophen as part of their pain medication treatment as opposed to 71% Austrian. Prescribed analgesics for all surgeries as well as combinations are shown in Fig. 1. NSAID therapy was given by 40% of Canadian surgeons, of which the majority (66%) gave ibuprofen. One surgeon prescribes naproxen and one other regularly prescribes toradol. NSAID therapy was part of the postoperative course in 76% in Austria, of which 84% included diclofenac, 54% ibuprofen and 12% naproxen. Mefenamic acid was reported separately from other NSAID therapies for better comparison since it was only prescribed in Austria and not in Canada.

**Fig. 2:**
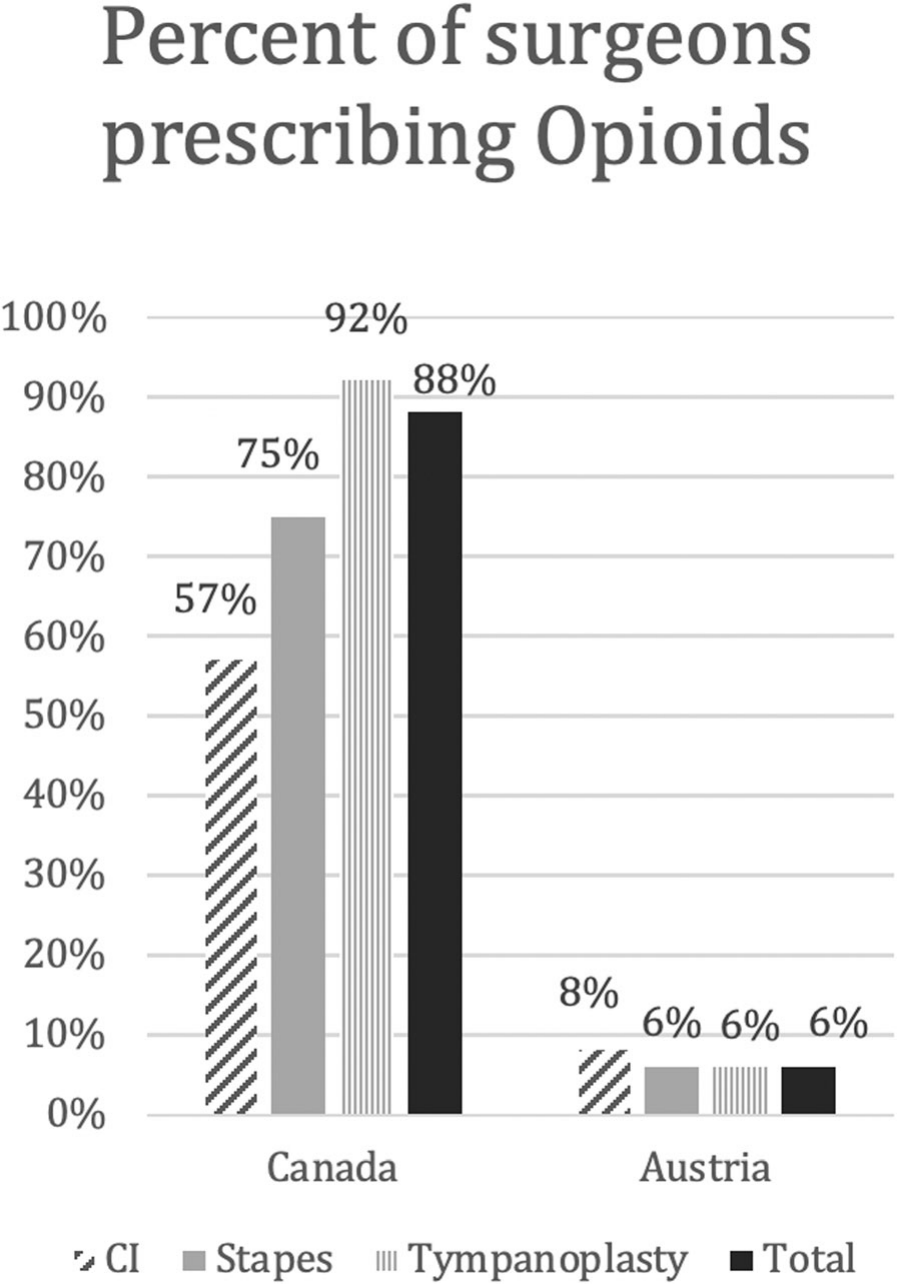
Opioids versus Non-Opioids. Percent of surgeons prescribing Opioids after certain surgeries (Cochlear Implant (CI), Stapedotomy (Stapes) and Tympanoplasty), as well as in total according to country (Canada and Austria

**Fig. 3:**
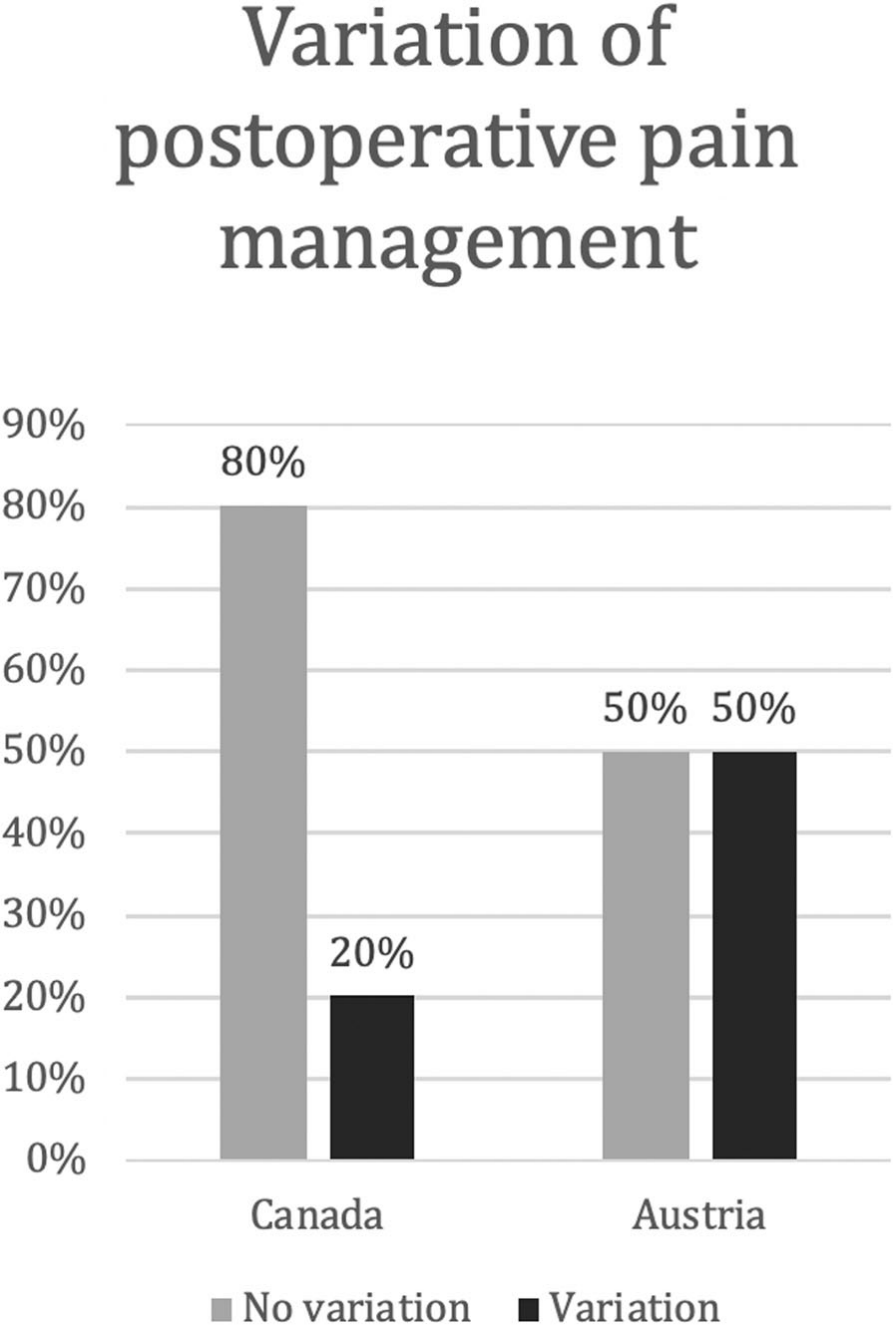
Variation of pain management. Percent of surgeons, who give the same pain medication for all three surgeries (no variation), Percent of surgeons, who give different pain medication for surgeries (Variation). The left columns show the results of the Canadian otologists, the right columns show the results of the Austrian otologists

Celecoxib and hydromorphone were not selected by any surgeon and therefore removed from analysis. The likelihood of surgeons prescribing any opioid for the three main surgery types are shown in Fig. 2. The results given by every single surgeon for different surgeries were further analyzed and categorized into two groups. Group 1 gave the same medication or group of medications for every surgery. Group 2 varied pain medication depending on the surgery. Some surgeons (*n* = 4) only performed one of the analyzed surgeries, so comparison was not possible. Results are depicted in Fig. 3.

Local anesthetic was used at the end of surgery for postauricular approaches with the goal of reducing immediate postoperative pain by five surgeons in total of which, four were Canadians. Of these local anesthetics, bupivacaine was routinely used by three surgeons, with the remaining two using lidocaine and rupivicaine.

### Confidence in pain management

**Fig. 4:**
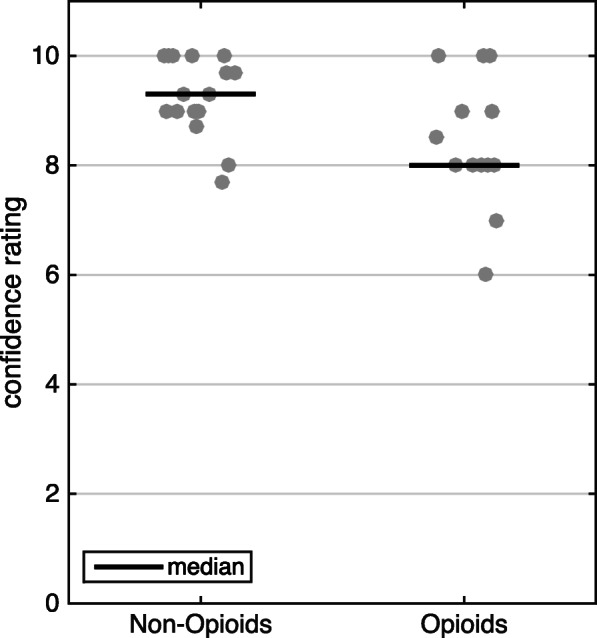
Confidence in pain medication. Each dot represents a surgeon’s mean confidence in the prescribed pain medication. Surgeons were grouped into two groups: Non-opioids - do not prescribe any opioids, Opioids – prescribe opioids for postoperative pain

Surgeons answering the questionnaires rated their confidence with the pain medication prescribed by them at the end of each subsection (cochlear implant surgery, stapedotomy and tympanoplasty). Overall confidence of Canadian surgeons was 8.33, and 9.43 amongst the Austrian surgeons. Canadians rated their pain management for cochlear implant surgery, stapedotomy and tympanoplasty to be 8.5, 8.3 and 8.2 effective on a scale from 0 to 10 for the three surgeries, respectively. The Austrian surgeons rated the pain medication for the same operations with 9.2, 9.6 and 9.5. The difference between the two groups was statistically significant *p* = 0.037, results are depicted in Fig. 4.

## Discussion

The purpose of this survey was to illustrate the substantial variability of prescribing patterns after otologic surgery between two countries (Canada and Austria). Only one Austrian surgeon regularly prescribes any opioid after otologic surgeries, as opposed to 88% of Canadian otologists.

Opioids are potent and effective for pain management, especially in the setting of chronic pain [12]. Many studies have been conducted evaluating this type of pain management. Béliveau et al. assessed patients using the Quebec administrative claims database without a cancer diagnosis [12]. Of the 124,664 patients, who had one course of opioid therapy, 4172 which equals 3.3% progressed to long-term users. Adjusted analysis found the following associated factors with long-term use in said study: prescription of acetaminophen-codeine, prescription of a long-acting opioid at initiation, initial supply of 30 days or more, chronic pain as well as initial dose of more than 90 morphine milligram equivalents. Studies, such as the one mentioned before, and others have shown that chronic opioid use often starts with the first use of these drugs for example with acute pain after surgery [13].

Intuitively, postoperative pain levels depend on the surgical site within otolaryngological procedures [14]. Otologic procedures are notably less painful than operations on the larynx or pharynx [14]. Consequently, recent studies have suggested incomplete consumption of prescribed opioids after otologic surgery [2, 10]. Since safe disposal of prescribed opioids is rarely performed, the potential for misuse and abuse may be inadvertently increased [4]. The literature on pain after otologic surgery and pain control with non-opioid medication is scarce. A recent systematic review concluded that the addition of codeine provides superior pain management than acetaminophen (i.e. Paracetamol) alone in otologic surgeries. The authors also stated that there might be a sufficient effect by NSAID or α-agonists monotherapy, as well as through nerve blocks [15], however, further studies assessing non-opioid use for pain control after otologic surgery is needed. Similar or even superior pain control of NSAIDs compared to opioids such as hydrocodone have been shown for various other surgical procedures such as rhinoplasties [16] or for acute pain in general, including postoperative pain [17].

In this survey-based study we discovered that 57 to 92% of Canadian otolaryngologists prescribe opioids for acute postoperative pain, depending on type of surgery. In contrast 93% of Canadian otolaryngologists prescribe Acetaminophen as opposed to 71% of Austrian otolaryngologists as part of their postoperative pain regimen. The reasons for these discrepancies are most likely innumerable and might include issues such as accessibility, advertising, education or cultural differences. There are no studies on non-opioid analgesia following middle ear surgery, which is why there is no evidence for this treatment course so far [15]. This could have led to the lower prescription of NSAIDs after ear surgery in Canada compared to Austria. Surgeons might fear increased risk of bleeding postoperatively by administering NSAIDs despite a recent meta-analysis of 27 randomized clinical trials refuting an increased risk of post-operative bleeding in patients treated with NSAIDs [18]. Further discrepancy of given pain medications in the presented study may be due to the use of metamizole, an NSAID alternative that was used by nearly a third of the Austrian otologists. This medication was withdrawn from the North American market in the 1970s following reports of fatal agranulocytosis [19]. In countries in which Metamizole is approved, it is often given additionally to NSAID therapy for short term pain control or as primary therapy for patients with kidney disease [20].

Overall, mainly weak opioids seem to be prescribed by otologists in these two countries. None of the Austrian and 20% of Canadians regularly prescribe strong opioids such as morphine or oxycodone. One of the most common prescribed opioids in this survey among Canadian otologists was Codeine. The problem with this specific weak opioid is that it’s efficacy is limited by its variable metabolism [21]. Codeine is a prodrug that undergoes O-demethylation into morphine via CYP2D6 [21]. CYP2D6 poor metabolizers have low plasma concentrations of morphine after taking codeine and probably do not have any clinical benefit [21]. In contrast, ultrarapid metabolizers of codeine have a risk of increased toxicity, especially in young users, resulting in apnea [21]. Although Codeine is a so called weak opioid, studies have shown that it is associated with misuse, dependence, and overdose-related mortality [22]. Additionally, as it is often combined with other medications such as Acetaminophen or Ibuprofen, studies have shown that misuse of these combinations lead to severe toxicities of the non-opioid medication due to overdoses [22].

A further interesting discovery of this study is that Acetaminophen is prescribed by almost all Canadian otologists as baseline pain medication as opposed to approximately 70% of Austrians. Studies often suggest combining Acetaminophen and NSAIDs provides synergistic pain control [18].

All surgeons have the responsibility to work at combating the excess prescription of opioids, especially since it is estimated that about 36% of all dispensed opioid medications are prescribed by surgeons in the U.S. [23]. With this study, we aim to draw attention to the fact that there are large discrepancies in postoperative pain prescription patterns and that surgeons from different countries can learn from each other especially when it comes to combating the opioid crisis.

Limitations or weaknesses of this study clearly include that it only assesses prescribing patterns of otolaryngologists and no patient data. Further the sample size of otolaryngologists only including two countries allows limited conclusions. We did not assess preoperative use of short or long lasting local anesthetics, as well as the pain medications used before, during or the end of anesthesia/surgery. Nevertheless, we believe that this questionnaire based analysis provides important aspects and insights to possibilities of non-opioid therapy for postoperative pain. As mentioned above, in several other surgical fields sufficient postoperative pain control with NSAIDs comparable or even superior to opioids has been shown in randomized studies and meta-analyses [16, 17, 24, 25]. Primarily, treatment of pain following otologic surgery should therefore be attempted without opioids.

## Conclusion

Prescribing patterns differ substantially between the two evaluated countries with a clear favor for opioids in Canada compared to Austria. Several potential barriers to reducing opioid prescriptions exist including cultural expectations of pain, availability of non-opioid alternatives, and potential adverse outcomes of NSAIDs. An opportunity to reduce opioid dependency exists and further investigations evaluating different analgesia protocols following otologic procedures.

## Data Availability

The datasets during and/or analysed during the current study available from the corresponding author on reasonable request.

## Abbreviations

CI: Cochlear Implant
NSAID: Non steroidal anti-inflammatory drugs
U.S: United States
